# Dietary and lifestyle inflammation scores in relation to colon cancer recurrence in subgroups of patients based on common molecular tumour characteristics

**DOI:** 10.1016/j.esmogo.2025.100202

**Published:** 2025-07-17

**Authors:** E. Wesselink, D.E. Kok, K.C. Smit, A.-S. van Lanen, J.W.G. Derksen, M. Koopman, M. Ligtenberg, I.D. Nagtegaal, P.D.M. Rombout, J.H.W. de Wilt, E. Kampman, A.M. May, F.J.B. van Duijnhoven

**Affiliations:** 1 Division of Human Nutrition and Health, Wageningen University & Research, Wageningen, The Netherlands; 2 Division of Molecular Pathology, Netherlands Cancer Institute—Antoni van Leeuwenhoek Hospital, Amsterdam, The Netherlands; 3 Julius Center for Health Sciences and Primary Care, University Medical Center Utrecht, Utrecht University, Utrecht, The Netherlands; 4 Department of Medical Oncology, University Medical Center Utrecht, Utrecht University, Utrecht, The Netherlands; 5 Department of Human Genetics, Radboud University Medical Center, University of Nijmegen, Nijmegen, The Netherlands; 6 Department of Pathology, Radboud University Medical Center, University of Nijmegen, Nijmegen, The Netherlands; 7 Department of Surgery, Radboud University Medical Center, University of Nijmegen, Nijmegen, The Netherlands

**Keywords:** lifestyle inflammation scores, dietary inflammation scores, colon cancer recurrence, molecular tumour characteristics

## Abstract

**Background:**

The aim of this study was to investigate associations between the inflammatory potential of diet and lifestyle in relation to colon cancer recurrence in subgroups of patients based on molecular tumour characteristics that also influence the inflammatory tumour microenvironment.

**Patients and methods:**

A nested case-control study was implemented in two prospective cohort studies of colon cancer patients. Participants who developed a recurrence were included as cases (*n* = 167). Matched controls (*n* = 668) were selected based on incidence density sampling. Lifestyle factors were assessed at diagnosis using self-administered questionnaires and dietary intake was assessed using a food frequency questionnaire. The dietary inflammation score (DIS) and the lifestyle inflammation score (LIS) were constructed. High-throughput next-generation sequencing of tumour tissue was used for mutation and microsatellite instability (MSI) analysis. Associations between the DIS and LIS and recurrence were assessed with conditional logistic regression analyses in all patients, as well as in subgroups based on MSI. For patients with microsatellite stable (MSS) tumours, further stratification based on mutation status of *KRAS, BRAF, PIK3CA, TP53* and *APC* was performed.

**Results:**

A more pro-inflammatory diet was not associated with risk of recurrence [incidence rate ratio (IRR) 1.04, 95% confidence interval (CI) 0.96-1.12]. Persons who have a more pro-inflammatory lifestyle may have an increased recurrence risk (IRR 1.21, 95% CI 0.97-1.52), which was most pronounced for persons with MSS and *KRAS* or *PIK3CA* wildtype tumours (IRR 1.31, 95% CI 0.90-1.90 and IRR 1.30, 95% CI 0.98-1.71, respectively).

**Conclusion:**

Our results suggest that associations between the LIS and recurrence might differ based on molecular tumour characteristics.

## Highlights

• Our study suggests a pro-inflammatory lifestyle, but not diet, is linked to higher risk of colon cancer recurrence.
• Associations seem to be most pronounced in microsatellite stable and *KRAS* or *PIK3CA* wildtype tumours.
• Our study offers insights into which patients may benefit most from a less pro-inflammatory lifestyle after colon cancer.

## Graphical abstract

Graphical abstract

**Figure:**
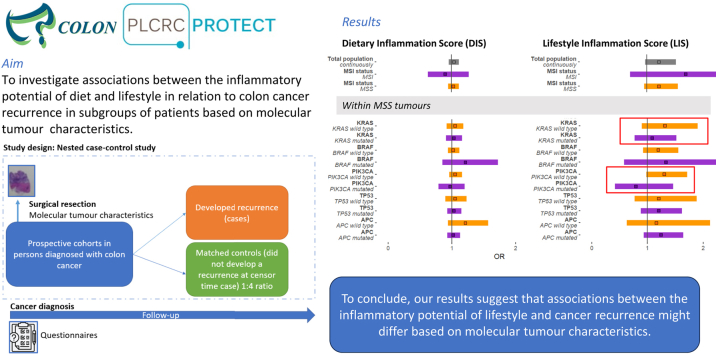

## Introduction

Inflammation is involved in every part of the cancer continuum, from development of the primary tumour to progression and recurrence.^1^^,^^2^ Inflammation plays a key role in the hallmarks and enabling characteristics of cancer, directly fostering tumour growth, inducing genome instability and mutations, and impairing the antitumour immune response.^2^, ^3^, ^4^, ^5^ Given the role of inflammation in tumour growth and development of metastasis, inhibiting inflammation could be a potential target to delay tumour recurrence.

Lifestyle factors, including diet and physical activity, could influence chronic inflammation.^6^^,^^7^ In several studies among colorectal cancer (CRC) survivors, a more pro-inflammatory diet, linked to higher levels of circulating inflammation markers, has been associated with an increased risk of CRC-specific mortality.^8^, ^9^, ^10^ In our previous research in the COLON study, we observed that a more pro-inflammatory diet, by means of a higher Empirical Dietary Inflammatory Pattern (EDIP) score, was associated with a higher risk of CRC recurrence.^11^

While chronic inflammation can induce mutations, several molecular tumour characteristics, including microsatellite instability (MSI) and driving mutations commonly observed in colon tumours, such as *KRAS* (30%-40%), *BRAF* (10%-20%), *PIK3CA* (20%-25%)*, TP53* (40%-50%) and *APC* (>80%),^2^^,^^5^^,^^12^^,^^13^ can also influence the inflammatory tumour environment and, subsequently, antitumour immune responses.^14^, ^15^, ^16^, ^17^ *KRAS* mutations, but also *TP53* mutations, are hypothesized to drive a pro-inflammatory tumour environment by activation of several inflammatory pathways, including the NF-kB, NLRP3 and STAT3 pathways, leading to immune evasion and treatment resistance.^12^^,^^14^^,^^16^^,^^18^^,^^19^ *PIK3CA-* and *BRAF*-mutated tumours generally have a pro-inflammatory and immune-suppressive profile, mainly through interactions with the COX-2 pathway and up-regulation of *PEG2*, leading to diminished immune response against the tumour.^20^, ^21^, ^22^ Patients with tumours that are less pro-inflammatory and more responsive to immune activation may respond differently to lifestyle-induced inflammation compared with tumours with a pro-inflammatory and immune-suppressive profile. Hence, a more anti-inflammatory lifestyle might be more beneficial for a subgroup of patients with a less inflammatory and immune-sensitive tumour.

The overall aim of this study was to evaluate the association between the inflammatory potential of the diet and other lifestyle factors and colon cancer recurrence in a subgroup of patients based on MSI and mutation status of *KRAS, BRAF, PIK3CA, TP53* and *APC*. Ultimately, insight into these associations in the presence or absence of specific molecular tumour characteristics could contribute to the development of personalized lifestyle guidelines for individuals diagnosed with colon cancer.

## Methods

### Study population

Both the COLON study (ClinicalTrials.gov Identifier NCT3191110) and the Prospective Dutch Colorectal Cancer (PLCRC)-PROTECT study (ClinicalTrials.gov Identifier NCT-02070146) have been described in detail previously.^23^, ^24^, ^25^ Briefly, in the COLON study, newly diagnosed CRC patients were recruited from 11 Dutch hospitals, from August 2010 until February 2020. Men and women >18 years with all stages of disease were eligible. Participants were not invited when unable to speak Dutch; or with a history of CRC, (partial) bowel resection or inflammatory bowel disease, inherited CRC syndrome (Lynch syndrome, familial adenomatous polyposis and Peutz–Jeghers syndrome); or with mental conditions such as dementia that make it difficult to fill out surveys. For the PLCRC-PROTECT study, newly diagnosed CRC patients were recruited from 21 Dutch hospitals starting from February 2016. Both studies were approved by a medical ethics committee (COLON: CMO region Arnhem-Nijmegen, 2009-349; PLCRC-PROTECT: University Medical Center Utrecht, 15-770). All study participants provided written informed consent.

### Nested case-control design

For the current analyses with colon cancer recurrence as the outcome, a nested case-control study was implemented in the COLON and PLCRC-PROTECT studies. Patients who received neoadjuvant treatment were not eligible for the nested case-control study, since tumour characteristics assessed in the surgical specimens may have been affected by this treatment. Because ∼70% of all rectal cancer patients received neoadjuvant treatment within the COLON and PLCRC-PROTECT studies, we specifically focussed on patients with stage I-III colon cancer^26^ who did not receive neoadjuvant treatment. In addition, patients with missing dietary data were excluded from this study. In total, 1555 participants were eligible for the nested case-control study (*n* = 1176 from the COLON study and *n* = 379 from the PLCRC-PROTECT study).

**Figure 1:**
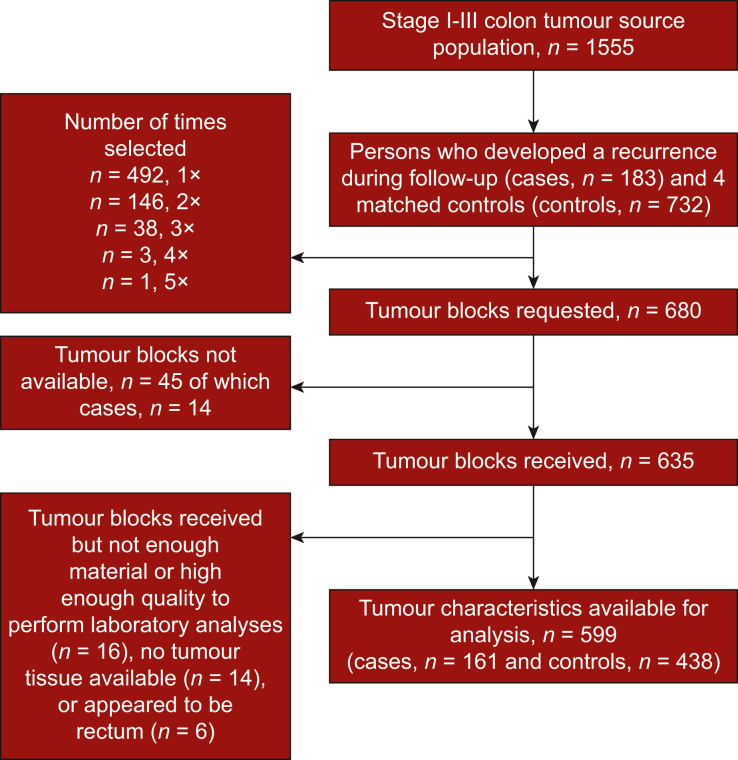
Flowchart of selection of tissue blocks.

During the follow-up of the COLON and PLCRC-PROTECT studies, 183 participants (*n* = 140 COLON; *n* = 43 PLCRC-PROTECT) diagnosed with stage I-III colon cancer developed a recurrence and were included as cases. The median follow-up time until the occurrence of an event (recurrence) was 1.7 years [inter quartile range (IQR) 1.2-3.3 years] in the cases. Recurrence was defined as a locoregional recurrence and/or distant metastasis. Information on colon cancer recurrence was collected through the medical records by the Netherlands Cancer Registry. Controls were selected based on incidence density sampling from all eligible participants alive and free of a colon cancer recurrence at the time of the recurrence of the matching case with a 4 : 1 control to case ratio. Cases and controls were matched on age (±10 years), sex (men, women), stage of disease (I, II, III) and hospital in which they were treated. Selection and matching were done for each cohort separately. When it was not possible to match four controls based on all these matching criteria, the hospital criterion was omitted, and remaining controls were selected based on age, sex and stage of disease. Since it was possible to be selected more than once as a control (Figure 1), 167 cases and 668 controls were selected in the nested case-control study, which corresponds to 680 tumour tissue blocks.

### Tumour tissue and laboratory analyses

Tissue blocks were obtained from the pathology archives to assess the molecular tumour characteristics. In The Netherlands, tissue specimens for all patients who received surgical resections are stored and registered in the Dutch nationwide pathology databank (PALGA).^27^ Of the 680 formalin-fixed and paraffin-embedded (FFPE) tissue blocks requested from 25 labs, 635 were sent to Radboudumc, Nijmegen, The Netherlands, for analyses. Of these FFPE tissue blocks, 621 could be used for analyses, and 14 could not be used because they did not have any available tumour tissue or contained noncolon tumour tissue. In the case of multiple tumour blocks per participant, tumour sections with the highest tumour percentage were selected for further analyses.

FFPE tissue sections were used to determine molecular tumour characteristics. Haematoxylin–eosin (H&E)-stained sections were used to semiquantify tumour percentage within the tissue and tumour tissue was isolated from surrounding healthy tissue by means of macrodissection. DNA of the tumour was isolated using a method based on Chelex, followed by ethanol precipitation. A comprehensive panel, the PATH gene panel (PATHv3D), established at the laboratory of pathology of the Radboudumc and encompassing regions in 47 relevant CRC-associated genes, including the oncogenes *KRAS, BRAF* and *PIK3CA,* and tumour suppressor gene *TP53,* was analysed.^28^ Additionally, the *APC* gene was sequenced at Radboudumc. The mutation status of these genes (PATH gene panel, as well as APC) was determined using high-throughput next-generation sequencing (NGS) on the Novaseq 6000 (NovaSeq 5000/6000 S1 Rgt Kit; Illumina, San Diego, CA). Demultiplexed FASTQ files were uploaded and analysed in Sequence Pilot version 5.2.0 (JSI Medical Systems, Ettenheim, Germany),^29^ which included removal of *PIK3CA* and *PTEN* pseudogene reads from the alignment and subsequent analysis.^28^ After variant calling, all variants with a variant allele frequency >3% were visually inspected in Sequence Pilot software, and curated and classified as benign, likely benign, variant of unknown significance, likely pathogenic, or pathogenic. Pathogenic and likely pathogenic variants were considered ‘mutated’. Additionally, 57 markers that were also sequenced using the PATHv3D panel were used to define MSI^28^^,^^30^ with mSINGS,^31^ in which ≥30% unstable loci is considered as MSI, ≤15% as microsatellite stable (MSS) and 15%-30% as uncertain. The PATH panel analyses were carried out in duplicate, the APC analyses in singleplex. The mutation status of included genes was evaluated by two assessors; in case of discrepancy consensus was reached by consulting a third independent assessor. Of the 621 analysed samples, 16 samples could not be used because the material was of insufficient quality to determine mutation status and MSI and 6 samples appeared to originate from rectal tissue instead of colon tissue, resulting in 599 valid samples for further data analyses (Figure 1).

### Data collection

In the COLON and PLCRC-PROTECT studies, information on demographics, smoking status, education, medication usage and self-assessed anthropometric measurements such as weight and height were collected through a self-administered questionnaire.^23^ A semiquantitative food frequency questionnaire (FFQ) of 204 items developed by the Division of Human Nutrition and Health of Wageningen University & Research was used to assess habitual dietary intake in the month before primary cancer diagnosis.^23^^,^^32^^,^^33^ Dietary supplement use was assessed in both cohorts using a self-administered dietary supplement questionnaire developed by the Division of Human Nutrition and Health of Wageningen University & Research, The Netherlands.^23^ Type of dietary supplements, frequency and duration of intake and dosage was recorded. Physical activity was assessed by the Short Questionnaire to ASsess Health-enhancing physical activity (SQUASH).^34^ The dietary inflammation score (DIS) and the lifestyle inflammation score (LIS) were used as proxy for the inflammatory potential of diet and lifestyle, respectively. In our previous work in the full COLON and PLCRC cohorts we assessed associations between inflammation markers and the DIS and LIS in the subpopulation for which inflammation marker data was available. A higher DIS seems to be associated with higher circulating levels of interleukin (IL)-6, IL-8 and high-sensitive C-reactive protein (hsCRP). A higher LIS was associated with higher circulating levels of IL-6, IL-8, tumour necrosis factor-α and hsCRP.^35^

### Dietary inflammation score components and calculation

The DIS consists of 19 items and was calculated as previously described by Byrd et al.^36^ Although the DIS was developed and validated in a United States population, it has been successfully applied in other settings before, such as European populations.^37^ The 18 food groups in the DIS are ‘leafy greens and cruciferous vegetables’, ‘tomatoes’, ‘apples and berries’, ‘deep yellow or orange vegetables and fruits’, ‘other fruits and real fruit juices’, ‘other vegetables’, ‘legumes’, ‘fish’, ‘poultry’, ‘red and organ meats’, ‘processed meats’, ‘added sugar’, ‘high-fat dairy’, ‘low-fat dairy’, ‘coffee and tea’, ‘nuts’, ‘other fats’, ‘refined grains and starch vegetables’. Next to the food groups, supplement use was also included in the score. For the dietary supplement component, a combined score of micronutrients was calculated. This component was obtained by first ranking single supplemental micronutrient intakes into tertiles, stratified by sex. Secondly, a value of 2 was assigned to the highest tertile, 1 to the middle and 0 to the lowest tertile. Thirdly, multiplication took place with a value of −1 for the pro-inflammatory micronutrient iron.^36^ Multiplication with a value of +1 took place for the anti-inflammatory micronutrients (vitamins A, B12, B6, C, D and E; β-carotene, folate, niacin, riboflavin, calcium, magnesium, selenium, thiamine and zinc).^36^ Finally, a total supplemental micronutrient intake score was derived by summing all the values of the single nutrients, where a higher supplement score corresponds to a higher intake of anti-inflammatory supplements. This total supplemental micronutrient intake score represents the supplement intake component within the total DIS.

Each component (18 food group components and one supplement intake component) of the DIS was standardized by sex to a mean of 0 and standard deviation of 1, to obtain z-scores. Consequently, all food groups were multiplied by their respective weight (see Table 2). Finally, the sum of the intake of food groups × weights constituted the overall DIS. A higher score means an exposure to a relatively more pro-inflammatory diet.

**Table 2:** Inflammatory weight and descriptives of the components of the dietary and lifestyle inflammation scores (DIS and LIS), stratified by cases and matched controls

		Cases	Matched controls
	Inflammatory weight		
***N***		167	668
**DIS**		0.33 (−1.23 to 1.87)	0.16 (−1.37 to 1.58)
**LIS**		0.23 (−0.18 to 0.89)	0.05 (−0.41 to 0.73)
**Unknown**		5	34
**Intake of food groups**^a^			
**Leafy greens and cruciferous vegetables**	−0.14	32 (18-48)	28 (16-45)
**Tomatoes**	−0.78	4 (1-12)	5 (2-14)
**Apples and berries**	−0.65	76 (36-119)	84 (38-150)
**Yellow vegetables and fruits**	−0.57	12 (5-21)	11 (5-20)
**Other fruits and real fruit juices**	−0.16	131 (63-214)	115 (53-195)
**Other vegetables**	−0.16	17 (7-32)	18 (9-34)
**Legumes**	−0.04	20 (8-36)	20 (9-32)
**Fish**	−0.08	9 (4-15)	10 (5-17)
**Poultry**	−0.45	10 (4-18)	11 (6-21)
**Red and organ meats**	0.02	38 (19-56)	38 (22-56)
**Processed meats**	0.68	23 (12-39)	24 (11-39)
**Products high in added sugars**	0.56	22 (7-62)	22 (8-56)
**High-fat dairy**^b^	−0.14	79 (42-162)	66 (32-121)
**Low-fat dairy**	−0.12	145 (60-257)	175 (64-291)
**Coffee and tea**	−0.25	580 (435-779)	580 (464-813)
**Nuts**	−0.44	6 (1-16)	9 (3-20)
**Other fats**	0.31	18 (9-30)	20 (10-29)
**Refined grains and starchy vegetables**	0.72	253 (190-322)	260 (196-322)
**Energy (kcal/day)**		1.775 (1.427-2.133)	1.827 (1.525-2.168)
**Any supplement use (yes)**	−0.80	65 (39)	326 (50)
**Unknown**		3	19
**Alcohol**		5 (0-20)	5 (0-20)
**Alcohol categorical**^c^			
**Heavy**	0.30	27 (16)	129 (19)
**Moderate**	−0.66	83 (49)	348 (52)
**None**		58 (35)	194 (29)
**Moderate to vigorous physical activity (hours/week)**		10 (5-20)	12 (5-21)
**Unknown**		5	33
**BMI**			
**Continuous**		27.0 (24.2-29.4)	25.9 (23.7-29.0)
**Normal (<25 kg/m^2^)**		58 (35)	277 (41)
**Overweight (25-30 kg/m^2^)**	0.89	37 (22)	119 (18)
**Obese (>30 kg/m^2^)**	1.57	73 (43)	274 (41)
**Unknown**		0	1
**Smoking status**			
**Current**	0.50	16 (9.6)	43 (6.5)
**Former and never**		151 (90)	620 (94)
**Unknown**		0	5

Values presented are median (Q1-Q3) or *n* (%).

BMI, body mass index.

a The intake of food groups is in g/day.

b Cream butter is included in the other fat food group.

c Alcohol intake, assessed by the food frequency questionnaire, was divided over the categories ‘none’ (<1 g/day), ‘moderate’ (>1 and ≤14 g/day for women and >1 and ≤28 g/day for men)’, or ‘heavy’ (>14 g/day for women and >28 g/day for men).

### LIS components and calculation

The LIS consists of four items: physical activity, smoking status, body mass index (BMI) and alcohol consumption.^36^ In the SQUASH questionnaire, participants were asked to report their average time per week spent on community activities, activity at work, household activities and leisure time activities. Physical activity level was quantified by assigning a metabolic equivalent of task (MET) value to each activity, using the Ainsworth compendium of physical activity, in combination with self-reported intensity.^34^ Tertiles of moderate-to-vigorous activity in hours per week were calculated. Smoking status was classified as current, former and never smokers. All former smokers stopped >1 year ago; if they stopped <1 year ago, they would be classified as current smokers. Self-assessed anthropometric measurements (height and weight) were used to calculate BMI (kg/m^2^). A BMI of ≤25 is considered ‘normal’, from >25 to <30 ‘overweight’ and ≥30 considered ‘obese’. Only seven people (1%) were underweight (BMI ranging between 17.4 and 18.4 kg/m^2^). Given the limited numbers, these people were categorized within the normal weight group for the construction of the score. Alcohol intake, assessed by the FFQ, was divided over the categories ‘none’ (<1 g/day), ‘moderate’ (>1 and ≤14 g/day for women and >1 and ≤28 g/day for men)’ or ‘heavy’ (>14 g/day for women and >28 g/day for men).

To calculate the LIS, all items were multiplied by their respective weight (see Table 2). Finally, the sum of the intake of lifestyle components × weights constituted the overall LIS. A higher score represented a more pro-inflammatory lifestyle.

### Clinical information

Clinical information regarding stage of disease, tumour location and presence of comorbidities was derived from the Dutch ColoRectal Audit for the COLON study^38^ or the Dutch Cancer Registry for the PLCRC-PROTECT study.

### Data analysis

Baseline characteristics presented for cases and matched controls are shown in Table 1. The data were described by medians and IQR, or percentages and frequencies. Table 2 shows the baseline descriptives of the DIS and LIS components, presented for cases and controls. The inflammatory weights of each item (derived from Byrd et al.^36^) are also included in this table.

**Table 1:** Baseline characteristics of colon cancer patients, stratified by cases and matched controls

	Cases, *n* (%)	Matched controls, *n* (%)
***N***	167	668
**Sex (female)**	58 (35)	221 (33)
**Age at diagnosis, years (median[Q1-Q3])**	67 (60-73)	66 (60-73)
**Education level**^a^		
**Low**	63 (38)	267 (40)
**Medium**	50 (30)	176 (27)
**High**	54 (32)	220 (33)
**Unknown**	0	5
**Regular use of NSAID**^b^		
**Yes**	17 (10)	62 (9.4)
**Unknown**	0	9
**ASA physical status classification**		
**1**	40 (25)	186 (29)
**2**	95 (59)	386 (60)
**3**	23 (14)	72 (11)
**4**	2 (1.3)	1 (0.2)
**Unknown**	7	23
**Location primary tumour**		
**Proximal colon**	75 (45)	297 (44)
**Distal colon**	92 (55)	371 (56)
**Stage of disease**		
**I**	10 (6.0)	47 (7.0)
**II**	51 (31)	200 (30)
**III**	106 (63)	421 (63)
**Type of treatment**		
**Only surgery**	71 (43)	291 (44)
**Adjuvant chemotherapy**	95 (57)	372 (56)
**Other**	1 (1)	5 (1)
**Tumour characteristics**		
**MSI**	15 (9)	85 (13)
**Unknown**	7	57
***KRAS* mutated**	77 (48)	246 (39)
**Unknown**	5	38
***BRAF* mutated**	29 (18)	104 (17)
**Unknown**	5	38
***TP53* mutated**	108 (67)	410 (65)
**Unknown**	5	38
***PIK3CA* mutated**	27 (17)	133 (21)
**Unknown**	5	38
***APC* mutated**	120 (76)	492 (80)
**Unknown**	9	54
**Cohort**		
**COLON**	128 (77)	521 (78)
**PLCRC**	39 (23)	147 (22)

Values presented are median (Q1-Q3) or *n* (%).

ASA, American Society of Anesthesiologists; BMI, body mass index; MSI, microsatellite instability; NSAID, nonsteroidal anti-inflammatory drug.

a Education: Low education is defined as primary school and general lower secondary school; medium as lower vocational training and higher general secondary education; high as high vocational training and university.

b Regular NSAID use: use of NSAID more than once a week.

An incidence rate ratio (IRR), which is the interpretation of an odds ratio in an incidence density sampling design,^39^ and 95% confidence interval (95% CI) for the associations between the DIS and LIS (continuously per one unit increase) and colon cancer recurrence were estimated using conditional logistic regression.^40^ These analyses were first stratified by MSI and MSS tumours (86%) and subsequently by other molecular tumour characteristics (i.e. *KRAS, BRAF, PIK3CA, TP53, APC* mutation status) (Table 3). As matching could introduce confounding,^41^ all matching variables (sex, age, disease stage) and cohort were included in the models. Although hospital was used as a matching variable in the COLON study, this was not added to the crude model a priori, because of the limited power. The effect of including hospital as a covariate into the model was tested in a sensitivity analysis. Also, energy intake was included in model 1. Model 2 was additionally adjusted for either lifestyle for the DIS model or diet for the LIS model. Adjusting for lifestyle was done by including the individual components of the LIS. Adjusting for diet was done by calculating an equally weighted DIS where, instead of the inflammatory weights from Table 2, either −1 (healthy food) or +1 (unhealthy food) was used. Additional potential confounders were added to the models when they changed the IRR substantially (>10%). Potential confounders considered were education level (low, medium, high), adjuvant chemotherapy (yes, no), daily nonsteroidal anti-inflammatory drug use (yes, no) and comorbidities (overall yes, no; cardiovascular diseases yes, no; diabetes yes, no; American Society of Anesthesiologists (ASA) score I, II, III, IV). None of these factors influenced the association and they were therefore not included in the models.

**Table 3:** Associations of the dietary and lifestyle inflammation scores (DIS and LIS) with recurrence in colon cancer patients, overall and by molecular tumour characteristics

		DIS	LIS
**Total population**	Recurrence (local-regional and metastasis)		
	N cases/controls	167/668	162/634
	Model 1^a^ IRR (95% CI)	1.05 (0.98-1.14)	1.23 (0.98-1.53)
	Model 2^b^ IRR (95% CI)	1.04 (0.96-1.12)	1.21 (0.97-1.52)
**Stratified microsatellite stability**			
**MSI**	N cases/controls	15/85	14/78
	Model 1 IRR (95% CI)	0.94 (0.71-1.26)	1.16 (0.56-2.42)
	Model 2 IRR (95% CI)	0.90 (0.63-1.27)	1.69 (0.70-4.01)
**MSS**	N cases/controls	145/526	142/504
	Model 1 IRR (95% CI)	1.05 (0.97-1.14)	1.22 (0.95-1.55)
	Model 2 IRR (95% CI)	1.03 (0.95-1.12)	1.21 (0.95-1.55)
**Further stratified for other mutations in MSS tumours**^c^			
***KRAS* mutated**	N cases/controls	74/225	72/212
	Model 1 IRR (95% CI)	1.03 (0.91-1.15)	1.08 (0.77-1.52)
	Model 2 IRR (95% CI)	1.03 (0.91-1.16)	1.08 (0.77-1.52)
***KRAS* wild type**	N cases/controls	69/297	68/288
	Model 1 IRR (95% CI)	1.09 (0.97-1.23)	1.32 (0.91-1.90)
	Model 2 IRR (95% CI)	1.05 (0.92-1.18)	1.31 (0.90-1.90)
***BRAF* mutated**	N cases/controls	16/44	16/42
	Model 1 IRR (95% CI)	1.12 (0.83-1.52)	1.32 (0.58-3.01)
	Model 2 IRR (95% CI)	1.21 (0.85-1.72)	1.33 (0.58-3.04)
***BRAF* wild type**	N cases/controls	127/478	124/458
	Model 1 IRR (95% CI)	1.04 (0.95-1.14)	1.20 (0.92-1.56)
	Model 2 IRR (95% CI)	1.02 (0.94-1.12)	1.19 (0.92-1.55)
**PIK3CA mutated**	N cases/controls	22/106	22/100
	Model 1 IRR (95% CI)	0.96 (0.78-1.18)	0.78 (0.42-1.44)
	Model 2 IRR (95% CI)	0.97 (0.79-1.20)	0.79 (0.42-1.46)
**PIK3CA wild type**	N cases/controls	121/416	118/400
	Model 1 IRR (95% CI)	1.07 (0.98-1.17)	1.30 (0.99-1.72)
	Model 2 IRR (95% CI)	1.05 (0.96-1.16)	1.30 (0.98-1.71)
**TP53 mutated**	N cases/controls	102/373	101/359
	Model 1 IRR (95% CI)	1.05 (0.95-1.17)	1.20 (0.89-1.62)
	Model 2 IRR (95% CI)	1.03 (0.93-1.15)	1.20 (0.88-1.62)
**TP53 wild type**	N cases/controls	41/149	39/141
	Model 1 IRR (95% CI)	1.06 (0.92-1.24)	1.21 (0.78-1.89)
	Model 2 IRR (95% CI)	1.05 (0.90-1.23)	1.20 (0.77-1.88)
**APC mutated**	N cases/controls	111/466	109/426
	Model 1 IRR (95% CI)	1.05 (0.96-1.15)	1.24 (0.94-1.64)
	Model 2 IRR (95% CI)	1.02 (0.93-1.13)	1.24 (0.93-1.64)
**APC wild type**	N cases/controls	28/62	27/60
	Model 1 IRR (95% CI)	1.20 (0.94-1.53)	1.15 (0.63-2.10)
	Model 2 IRR (95% CI)	1.21 (0.94-1.57)	1.16 (0.63-2.12)

Associations were assessed using conditional logistic regression analyses. DIS and LIS were added to the models as continuous variables, so IRRs are reflecting associations for a one unit increase in the DIS or LIS.

CI, confidence interval; IRR, incident rate ratio; MSS, microsatellite stable.

a Model 1 is adjusted for the matching factors; age at diagnosis (years), sex (male, female), stage of disease (I, II, III) and cohort (COLON/PLCRC), as well as for energy intake.

b Model 2 additionally adjusted for smoking status (current, former and never), physical activity level (hours/week moderate to vigorous activity), body mass index (kg/m^2^) and alcohol intake (g/day) for the DIS and diet quality (equally weighted DIS) for the LIS. The cases/controls of model 2 are equal to the cases/controls for the LIS model 1 models.

c Given the low number of microsatellite instable (MSI) tumours, we could not further stratify in MSI tumours.

To investigate whether observed associations might be different for locoregional recurrences versus distant metastases, persons with a locoregional recurrence (*n* = 40) were excluded in a sensitivity analysis.

As supplementary analyses, associations between tumour characteristics and recurrences were investigated using conditional logistic regression models. Similar as for the associations between DIS and LIS and recurrence, the matching factors sex, disease stage and cohort were included in all models. Also treatment with adjuvant chemotherapy (yes/no) was included in the model a priori, because of its influence on recurrence risk and since molecular tumour characteristics seem to be associated with chemotherapy efficacy.^42^^,^^43^

### Ethics approval

Both studies were performed in line with the principles of the Declaration of Helsinki and were approved by a medical ethics committee (COLON: region Arnhem-Nijmegen, 2009-349; PLCRC-PROTECT: University Medical Center Utrecht, 15-770/C). All study participants provided written informed consent.

## Results

### Baseline characteristics

In total, 167 cases and 668 matched controls were included in our data analyses. Cases and controls were successfully matched on age, sex and stage of disease (Table 1). In both groups, the medium age was 66 years (IQR 60-73 years), 33%-35% was female, and most participants had stage III disease at diagnosis (63%), followed by stage II (30%-31%) and stage I (6%-7%).

Compared with controls, cases had a slightly higher pro-inflammatory diet and lifestyle score (Table 2). In addition, cases had a higher intake of high-fat dairy and a lower intake of low-fat dairy and less often used dietary supplements. Cases had a higher BMI and were more often current smokers compared with matched controls (Table 2).

### The inflammatory potential of diet and lifestyle in relation to recurrence in subgroups of patients based on molecular tumour characteristics

A higher DIS, corresponding to a more pro-inflammatory diet, was not associated with the risk of colon cancer recurrence (IRR 1.04, 95% CI 0.96-1.12), while a higher LIS, corresponding to a more pro-inflammatory lifestyle, seemed to be associated with a higher risk of recurrence (IRR 1.21, 95% CI 0.97-1.52) in the overall study population (Table 3).

Although no statistically significant associations were found, several intriguing trends were observed. Null associations between the DIS and recurrence seemed to be consistent within all subgroups based on tumour characteristics. No differences in association between the LIS and recurrence were observed in subgroups of patients with an MSI versus an MSS tumour. Interestingly, association between LIS and recurrence seemed to be different in MSS *PIK3CA* wildtype (IRR 1.30, 95% CI 0.98-1.71) versus MSS *PIK3CA*-mutated tumours (IRR 0.79, 95% 0.42-1.46). Furthermore, associations between the LIS and recurrence seemed to be stronger in patients with an MSS *KRAS* wildtype tumour compared with patients with an MSS *KRAS*-mutated tumour (IRR 1.31, 95% CI 0.90-1.90 and IRR 1.08, 95% CI 0.77-1.52, respectively). Associations between the LIS and recurrence were similar in subgroups of MSS *BRAF* wildtype versus mutated*, TP53* wildtype versus mutated and *APC* wildtype versus mutated tumours.

In sensitivity analyses with colon cancer metastasis as outcome, associations seemed to be slightly stronger compared with the associations with locoregional recurrences and metastasis combined (Supplementary Table S1, available at https://doi.org/10.1016/j.esmogo.2025.100202). For example, for the MSS *PIK3CA* wildtype, a higher LIS was statistically significantly associated with a higher risk of colon cancer metastasis (IRR 1.48, 95% CI 1.09-2.00) compared with for the risk of locoregional recurrences and metastasis combined (IRR 1.30, 95% CI 0.98-1.71).

### Supplementary analyses: tumour characteristics and recurrence

MSI compared with MSS tumours seemed to be associated with a lower risk of colon cancer recurrence (IRR 0.60, 95% CI 0.33-1.09) (Supplementary Table S2, available at https://doi.org/10.1016/j.esmogo.2025.100202). Within MSS tumours, a tumour with a *KRAS* mutation compared with a *KRAS* wildtype seemed to be associated with a higher risk of colon cancer recurrence (IRR 1.40, 95% CI 0.96-2.04). Within MSS tumours, an *APC* mutation compared with an *APC* wildtype was associated with a lower risk of recurrence (IRR 0.56, 95% CI 0.34-0.91). No associations were observed between mutation status of *BRAF*, *PIK3CA* and *TP53* and colon cancer recurrence.

## Discussion

Our study is the first study to investigate associations between the inflammatory potential of diet and lifestyle in relation to colon cancer recurrence in subgroups based on common molecular tumour characteristics. A more pro-inflammatory diet was not associated with recurrence in the overall study population, nor in different subgroups based on molecular tumour characteristics. Persons with a more pro-inflammatory lifestyle seemed to have a higher risk of recurrence, although the association did not reach statistical significance. Associations between the LIS and recurrence appeared to be stronger in persons with an MSS *KRAS* or MSS *PIK3CA* wildtype tumour, compared with an MSS *KRAS-* or MSS *PIK3CA*-mutated tumour, respectively.

A more pro-inflammatory lifestyle, but not a more pro-inflammatory diet, seemed to be associated with a higher risk of recurrence in this nested case-control study. This is in line with our previous work in the overall COLON and PLCRC cohorts, where we observed a nonstatistically significantly higher risk of recurrence with a higher LIS (HR 1.10, 95% CI 0.91-1.35), but not with a higher DIS (0.95, 95% CI 0.89-1.02) in 2738 persons with stage I-III CRC.^35^ Since the COLON and PLCRC cohorts were the first studies in which associations between diet and lifestyle inflammation scores and colon cancer recurrence were investigated, our results should be confirmed in future studies.

Our results provide indications that associations between the LIS and colon cancer recurrence might be stronger in persons with *KRAS* or *PIK3CA* wildtype tumours. This would mean that adapting a less pro-inflammatory lifestyle might be especially beneficial for patients with a *KRAS* wildtype and/or *PIK3CA* wildtype tumour. While *KRAS* mutations are hypothesized to drive a pro-inflammatory tumour environment, *KRAS* wildtype tumours have a less aggressive inflammatory profile, leading to an environment that promotes immune activation against the tumour.^14^^,^^16^^,^^18^ Therefore, we hypothesize that *KRAS* wildtype tumours, compared with tumours with a *KRAS* mutation, might be more sensitive to the effects of lifestyle-induced inflammation. A similar mechanism can be hypothesized for *PIK3CA*-mutated versus wildtype tumours. *PIK3CA*-mutated tumours have a more pro-inflammatory and immune-suppressive profile, compared with *PIK3CA* wildtype tumours.^20^ As with *KRAS* wildtype tumours, *PIK3CA* wildtype tumours might better engage the immune system.^20^ Patients whose tumours are more sensitive to immune activation may be more likely to benefit from adopting a less pro-inflammatory lifestyle. In contrast, when the tumour microenvironment is predominantly immunosuppressive, it may override the potential beneficial effects of anti-inflammatory dietary and lifestyle changes. This suggests that the impact of lifestyle factors on recurrence could be modulated by tumour-intrinsic immune characteristics. An anti-inflammatory lifestyle includes becoming more physically active, reducing alcohol consumption, achieving a healthy BMI and stopping smoking. These lifestyle changes are not only potentially beneficial for cancer-related outcomes, but can also improve overall health and lower the disease burden of other chronic diseases, such as cardiovascular diseases, which are common in cancer survivors.^44^^,^^45^

In this study, association between tumour characteristics and recurrence, although not always statistically significant, was generally in line with results of previous studies.^43^^,^^46^, ^47^, ^48^, ^49^, ^50^, ^51^, ^52^ Associations between specific mutations and colon cancer recurrence often rely on the presence of co-existing mutations.^53^^,^^54^ However, analysing combinations of tumour mutations, especially within the context of lifestyle factors and recurrence, requires larger study populations.

More research is needed before recommendations regarding lifestyle based on molecular tumour characteristics can be provided. Firstly, our findings should be replicated in other study populations. Considering that analysing molecular tumour characteristics is both time consuming and costly, leveraging existing studies or datasets would be a preferable approach. Secondly, a larger population would allow for more robust analyses of how lifestyle interacts with combinations of mutations and other factors, such as cancer treatment, immune-related tumour characteristics such as immune cell infiltration and expression of inflammatory markers and germline genetics in the context of colon cancer recurrence. This level of detail could help refine personalized therapy approaches, including but not limited to lifestyle interventions, and could hopefully result in better patient outcomes.

This study stands out due to its comprehensive approach, integrating data on lifestyle factors, tumour characteristics and disease outcomes. However, this study is not without limitations. Although our study was relatively large, most of our findings did not reach statistical significance and should be interpreted with caution. Given our sample size, we would be able to detect an effect size (IRR) between 1.5 and 3.8 depending on the prevalence of the specific mutation with a power of 80% and an alpha of 0.05. Nevertheless, our results provide a valuable foundation for generating hypotheses and guiding future research. Furthermore, as the DIS and LIS were developed in a United States population, dietary intake and inflammatory weights might be different in our population of colon cancer patients in The Netherlands. We did observe an association between the EDIP score and cancer recurrence in our previous work within the COLON study.^11^ The EDIP score is a data-driven score, where inflammatory weights of food groups were derived from associations with inflammation markers in our own population of colon cancer patients. Therefore, the EDIP score, compared with the DIS, may better reflect the inflammatory potential of the diet for our specific study population. However, comparison with other studies and replication of results is easier with a standardized score such as the DIS. The associations observed between the DIS and LIS and recurrence in our nested case-control study were consistent with those in the overall COLON and PLCRC cohorts, while associations between molecular tumour characteristics and recurrence were mostly in line with previous literature, suggesting our nested case-control study accurately represents stage I-III colon cancer patients. Nevertheless, the generalizability of these findings to populations other than stage I-III colon cancer with a Western type of diet and lifestyle might be limited. Finally, investigating changes over time would be potentially interesting; based on our previous research, however, we observed that scores reflecting the inflammatory potential of diet and lifestyle remained relatively stable over time.^35^ Moreover, in longitudinal models within the full cohort, incorporating a slope to account for changes in these scores over time did not improve model performance,^35^ suggesting that baseline dietary patterns may be representative of the relevant exposure window for recurrence risk. In addition, the majority of cancer recurrences in our cohort occurred within the first 2 years after diagnosis (63%). Therefore, dietary intake 2 years after diagnosis would not be relevant to consider, since the exposure (dietary intake) in these cases is measured after the outcome (recurrent event).

To conclude, our results suggest that associations between the inflammatory potential of lifestyle and cancer recurrence might differ based on common molecular tumour characteristics. Larger studies, facilitated by consortia, are needed to further establish the interactions between lifestyle, tumour characteristics and colon cancer recurrence.
