# Supplementary material for: Dietary and lifestyle inflammation scores in relation to colon cancer recurrence in subgroups of patients based on common molecular tumour characteristics
Source: ESMO Gastrointest Oncol. 2025 Jul 17;9:100202. doi: 10.1016/j.esmogo.2025.100202 (PMC12836590; doi:10.1016/j.esmogo.2025.100202)
Supplement: Supplementary Tables 1 and 2 [file mmc1.pdf]

# Supplementary results for manuscript entitled: “Dietary and lifestyle inflammation scores in relation to colon cancer recurrence in subgroups of patients based on common molecular tumour characteristics.”

ESMO Gastrointestinal Oncology

Authors: Evertine Wesselink[1,2], Dieuwertje E. Kok[1], Karel C. Smit[3,4], Anne-Sophie van Lanen[1], Jeroen W.G. Derksen[3], Miriam Koopman[4], Marjolijn Ligtenberg[5], Iris D. Nagtegaal[6], Paul D.M. Rombout[6], Johannes H.W. de Wilt[7], Ellen Kampman[1], Anne M. May[3], Fränzel J.B. van Duijnhoven[1].

[1] Division of Human Nutrition and Health, Wageningen University & Research, Wageningen, The Netherlands;

[2] Division of Molecular Pathology, Netherlands Cancer Institute-Antoni van Leeuwenhoek Hospital, Amsterdam, The Netherlands;

[3] Julius Center for Health Sciences and Primary Care, University Medical Center Utrecht, Utrecht University, Utrecht, The Netherlands;

[4] Department of Medical Oncology, University Medical Center Utrecht, Utrecht University, Utrecht, The Netherlands;

[5] Department of Human Genetics, Radboud university medical center, University of Nijmegen, Nijmegen, The Netherlands;

[6] Department of Pathology, Radboud university medical center, University of Nijmegen, Nijmegen, The Netherlands;

[7] Department of Surgery, Radboud university medical center, University of Nijmegen, Nijmegen, The Netherlands.

**Table S1: Associations of the dietary and lifestyle inflammation score (DIS and LIS) with metastasis in colon cancer patients, overall and by molecular tumour characteristics.**

| | | DIS | LIS |
|---|---|---|---|
| ***Total population*** | *Metastasis* | | |
| | N cases/controls | 127/657 | 122/623 |
| | Model 1 IRR[a] (95% CI) | 1.03 (0.95 - 1.12) | 1.20 (0.93 - 1.54) |
| | Model 2 IRR[b] (95% CI) | 1.02 (0.94 - 1.11) | 1.21 (0.94 - 1.55) |
| **Stratified by microsatellite instability** | | | |
| **MSI** | N cases/controls | 6/81 | 5/74 |
| | Model 1 IRR (95% CI) | 0.84 (0.57 - 1.23) | 0.49 (0.13 - 1.83) |
| | Model 2 IRR (95% CI) | 0.85 (0.45 - 1.64) | 0.80 (0.15 - 4.18) |
| **MSS** | N cases/controls | 114/519 | 11/497 |
| | Model 1 IRR (95% CI) | 1.04 (0.95 - 1.13) | 1.27 (0.97 - 1.67) |
| | Model 2 IRR (95% CI) | 1.02 (0.93 - 1.11) | 1.27 (0.97 - 1.67) |
| **Further stratified for other mutations in MSS tumours[c]** | | | |
| **KRAS mutated** | N cases/controls | 61/225 | 59/212 |
| | Model 1 IRR (95% CI) | 1.00 (0.89 - 1.14) | 1.15 (0.80 - 1.66) |
| | Model 2 IRR (95% CI) | 1.01 (0.89 - 1.15) | 1.16 (0.80 - 1.67) |
| **KRAS wild type** | N cases/controls | 53/290 | 52/281 |
| | Model 1 IRR (95% CI) | 1.07 (0.93 - 1.22) | 1.40 (0.93 - 2.11) |
| | Model 2 IRR (95% CI) | 1.03 (0.90 - 1.18) | 1.40 (0.93 - 2.12) |
| **BRAF mutated** | N cases/controls | 12/42 | 12/40 |
| | Model 1 IRR (95% CI) | 1.29 (0.86 - 1.93) | 1.33 (0.52 - 3.43) |
| | Model 2 IRR (95% CI) | 1.35 (0.86 - 2.14) | 1.33 (0.51 - 3.46) |
| **BRAF wild type** | N cases/controls | 102/473 | 99/453 |
| | Model 1 IRR (95% CI) | 1.01 (0.92 - 1.11) | 1.29 (0.97 - 1.72) |
| | Model 2 IRR (95% CI) | 0.99 (0.90 - 1.09) | 1.30 (0.97 - 1.73) |
| **PIK3CA mutated** | N cases/controls | 15/106 | 15/100 |
| | Model 1 IRR (95% CI) | 0.90 (0.72 - 1.12) | 0.48 (0.21 - 1.11) |
| | Model 2 IRR (95% CI) | 0.93 (0.72 - 1.18) | 0.49 (0.21 - 1.14) |
| **PIK3CA wild type** | N cases/controls | 99/409 | 96/393 |
| | Model 1 IRR (95% CI) | 1.05 (0.95 - 1.17) | 1.48 (1.09 - 2.00) |
| | Model 2 IRR (95% CI) | 1.03 (0.94 - 1.14) | 1.48 (1.09 - 2.00) |
| **TP53 mutated** | N cases/controls | 82/366 | 81/352 |
| | Model 1 IRR (95% CI) | 1.02 (0.92 - 1.14) | 1.25 (0.90 - 1.74) |
| | Model 2 IRR (95% CI) | 1.01 (0.91 - 1.13) | 1.26 (0.91 - 1.75) |
| **TP53 wild type** | N cases/controls | 32/149 | 30/141 |
| | Model 1 IRR (95% CI) | 1.06 (0.90 - 1.25) | 1.32 (0.80 - 2.17) |
| | Model 2 IRR (95% CI) | 1.04 (0.88 - 1.22) | 1.30 (0.79 - 2.15) |
| **APC mutated** | N cases/controls | 90/441 | 88/421 |
| | Model 1 IRR (95% CI) | 1.02 (0.92 - 1.12) | 1.26 (0.93 - 1.71) |
| | Model 2 IRR (95% CI) | 1.00 (0.90 - 1.10) | 1.27 (0.93 - 1.73) |
| **APC wild type** | N cases/controls | 21/60 | 27/60 |

| | | | |
|---|---|---|---|
| | Model 1 IRR (95% CI) | 1.21 (0.93 - 1.58) | 1.52 (0.75 - 3.11) |
| | Model 2 IRR (95% CI) | 1.23 (0.93 - 1.63) | 1.56 (0.75 - 3.21) |

Associations were assessed using conditional logistic regression. DIS and LIS were added to the models as continuous variables, so IRRs are reflecting associations for a one unit increase in the DIS or LIS.

CI, confidence interval; IRR, incident rate ratio; MSS, microsatellite stable.

[a]Model 1 is adjusted for the matching factors; age at diagnosis (years), sex (male, female), stage of disease (I, II, III), and cohort (COLON/PLCRC), as well as for energy intake.

[b]Model 2 additionally adjusted for smoking status (current, former and never), physical activity level (hours/week moderate to vigorous activity), body mass index (kg/$m^2$) and alcohol intake (g/day) for the DIS and diet quality (equally weighted DIS) for the LIS. The cases / controls of model 2 are similar to the cases / controls for the LIS model 1 models.

[c]Given the low number of microsatellite instable (MSI) tumours, we could not further stratify in MSI tumours.

**Table S2: Associations of molecular tumour characteristics with colon cancer recurrence**

| | **Recurrence** | |
|---|---|---|
| **MSI status** | | |
| | **MSS** | **MSI** |
| N cases/controls | 145/526 | 15/85 |
| Adjusted IRR[a] (95% CI) | Ref | 0.60 (0.33 - 1.09) |
| **Other mutations in MSS tumours[b]** | | |
| | **KRAS wild type** | **KRAS mutated** |
| N cases/controls | 69/297 | 74/225 |
| Adjusted IRR (95% CI) | Ref | 1.40 (0.96 - 2.04) |
| | **BRAF wild type** | **BRAF mutated** |
| N cases/controls | 127/478 | 16/44 |
| Adjusted IRR (95% CI) | Ref | 1.33 (0.72 - 2.45) |
| | **PIK3CA wild type** | **PIK3CA mutated** |
| N cases/controls | 121/416 | 22/106 |
| Adjusted IRR (95% CI) | Ref | 0.71 (0.43 - 1.17) |
| | **TP53 wild type** | **TP53 mutated** |
| N cases/controls | 41/149 | 102/373 |
| Adjusted IRR (95% CI) | Ref | 1.01 (0.67 - 1.53) |
| | **APC wild type** | **APC mutated** |
| N cases/controls | 28/62 | 111/446 |
| Adjusted IRR (95% CI) | Ref | 0.56 (0.34 - 0.91) |

Associations were assessed using conditional logistic regression analyses.
CI, confidence interval; IRR, incident rate ratio; MSS, microsatellite stable.
[a]Models were adjusted for age at diagnosis (years), sex (male, female), stage of disease (I, II, III), adjuvant chemotherapy (yes/no) and cohort (COLON/PLCRC).
[b]Given the low number of microsatellite instable (MSI) tumours, we could not further stratify in MSI tumours.
